# Editorial: Job integration/reintegration of people with neuromuscular disorders in the epoch of “industry 4.0”

**DOI:** 10.3389/fneur.2024.1371430

**Published:** 2024-02-21

**Authors:** Alberto Ranavolo, Arash Ajoudani, Vincent Bonnet, Alessandro Marco De Nunzio, Francesco Draicchio, Massimo Sartori, Mariano Serrao

**Affiliations:** ^1^Department of Occupational and Environmental Medicine, Epidemiology and Hygiene, INAIL - National Institute for Insurance Against Accidents at Work, Rome, Italy; ^2^HRI2 Laboratory, Italian Institute of Technology (IIT), Genova, Italy; ^3^LAAS-CNRS, Université Paul Sabatier, CNRS, Toulouse, France; ^4^Research and Development Department, Lunex University, Differdange, Luxembourg; ^5^Department of Biomechanical Engineering, University of Twente, Enschede, Netherlands; ^6^Department of Medical and Surgical Sciences and Biotechnologies, Sapienza University of Rome, Latina, Italy

**Keywords:** job reintegration, industry 4.0, biomechanic analysis, neurological disorders, return to work

## Introduction

Individuals with neuromuscular disorders may have impairment in a number of motor functions, including reaching, grasping, balancing, and locomotion, during the working years (1–3). Rehabilitation, medical and surgical treatments, as well as ergonomic interventions, are all necessary to enable these people reintegrate into the workforce. It has been demonstrated that looking for a suitable job and avoiding premature exit from it improves the overall quality of life of these workers (1, 4–6). This is because these individuals were able to fully benefit from the positive effects of vocational rehabilitation and overcome barriers to finding, staying, or going back to employment (7–13). The ability of these people to maintain an effective motor behavior by adopting diverse compensatory strategies throughout the course of the disease, despite a possible illness progression and movement decline, supports this idea (14).

The European Union (EU) Equality Strategy for the Rights of Persons with Disabilities 2021–2030 states that the digital transformation is providing new opportunities to build on-site, and remote services suited to the requirements of people with disabilities using, among the others, artificial intelligence (AI) and human-robot collaboration (HRC) technologies. Therefore, extra ergonomic options afforded by technologies of the fourth industrial revolution should be considered when designing and developing reasonable and efficient job integration/reintegration programs for people with neuromuscular disability. Miniaturized wearable monitoring sensors (WS), feedback devices (FD), exoskeletons and collaborative robots (cobots) can be employed for movement-related elements. MS should be able to continuously decode and classify residual motor function in workers, monitor pre-post job integration programs and optimize HRC technologies controlling (15, 16). FD should be used for alerting purposes (17–20). HRC technologies should dynamically adapt to the workplace to aid disabled persons with a wide range of lower and upper limbs and trunk motor functions (21, 22).

## Return-to-work rehabilitation

Job integration of people with neuromuscular disorders is represented by any action taken to change or modify the job requirements, motor executions and work environment while taking into account training initiatives and technological advancements, such as HRC technologies, WS, FD, and AI), and the worker's clinical treatment outside the work context.

In this regard, a mini systematic review (Agostini et al.) has been published emphasizing the significance of including rehabilitation data when formulating return-to-work approaches. Particular focus should be made on the most practical ways for a better and quicker “restitutio ad integrum” of the worker. Motor imagery is considered a promising rehabilitation technique to reduce fatigue symptoms.

Findings of another study demonstrate that embodied rehabilitation improves body image perception, interoceptive awareness, balance, and quality of life (Paolucci et al.) which also reflects positively on the employment sphere.

To achieve effective job placement, it is essential to explore all therapeutic opportunities, including the proven Chinese massage as a feasible treatment for Peripheral neuropathy (Ge et al.).

## The European regulatory framework for job accommodation as a reference

In WHO European Region Member States approximately 6 to 10 individuals out of every 100 persons live with a disability. Persons with disabilities are affected by a low employment rate of around 50% and leave labor markets earlier. Article 1 of Council Directive 2000/78/EC of 27 November 2000 has the general provision to lay down a general framework for combating, among the others, discrimination on the grounds of disability. Article 7 states that “the principle of equal treatment shall be without prejudice to the right of Member States to maintain or adopt provisions on the protection of health and safety at work or to measures aimed at creating or maintaining provisions or facilities for safeguarding or promoting their integration into the working environment.” Article 26 of the Charter of Fundamental Rights of the EU states that “the EU recognizes and respects the right of persons with disabilities to benefit from measures designed to ensure their independence, social and occupational integration and participation in the life of the community.” The EU is engaged in improving the social and economic status of persons with disabilities based on the “Treaty on the Functioning of the EU.” The EU are party to the “United Nations Convention on the Rights of Persons with Disabilities (UNCRPD),” a treaty which has guided the contents of the “European disability strategy 2010–2020,” a renewed commitment to a barrier-free Europe. In the present decade, quality employment services are essential for all people with disabilities to live a dignified life (Equality Strategy for the Rights of Persons with Disabilities 2021–2030).

## Identifying the most appropriate technologies for movement monitoring and alerting to help people with neuromuscular disorders return to work

To analyze workers residual abilities, workstations, work environments, rehabilitation interventions and work gestures, kinematic, kinetic, and low- and high-density surface electromyographic data need to be collected.

Currently, workers are assessed using wearable, wireless, and miniaturized technology while doing manual material handling tasks (15, 16, 23, 24). Bipolar and high-density surface electromyography are used to estimate muscle behavior, insoles and sensorized shoes are used to measure reaction forces (kinetics), and inertial measurement units (IMUs) and 3D depth cameras are utilized for whole-body 3D reconstructions (kinematics) (15, 16, 23).

These wearables represent an effective way to control worker centered occupational HRC technology. It has also been shown that these sensors can deliver auditory, visual, and haptic-vibrotactile stimuli (25) representing promising solutions to lower biomechanical effort and injuries related to the workplace.

Industry 4.0 has made innovative HRC technologies more broadly available, and they are successfully supporting people in the workplace in real time. Miniaturized WS are essential in this context for the classification of residual motor functions and optimization of human-robot interfaces.

## Determining the most relevant indices for motor/muscle performance monitoring

In a systematic review (Chini et al.) that was published in this article collection, efficient indicators for workers monitoring in the occupational integration/reintegration period has been very thoroughly covered. The authors draw attention to the numerous quantitative physiological and biomechanical indices proposed in the literature. Compared to those produced from kinetic and sEMG measurements, those obtained from kinematic measurements are more commonly used (15). These indices make it possible to effectively evaluate ergonomic interventions for workplace and work task rehabilitation effectively, enabling accurate motor monitoring in work integration/reintegration programs. Many of these indices also have modest processing costs, making them ideal for real-time HRC technology monitoring applications.

Assessing the new human-robot interfaces (HRI) paradigm to promote re-integration of individuals who have suffered from neuromuscular injuries, such as stroke, muscle-tendon ripping, and musculoskeletal discomfort.

A potential alternative to the traditional assistive system to promote the re-integration of individuals who have suffered from neuromuscular injuries would involve an independent robotic system that acts as a supernumerary body capable of applying external forces to the patient, emulating the assistance that therapist would provide. According to the preliminary work presented by Ruiz-Ruiz and colleagues (26), collaborative mobile manipulators seem to be a workable and promising solution. The authors proposed the usage of a mobile collaborative robot with an interaction-assistive whole-body interface to help people unable to maintain balance.

## Author contributions

AR: Writing—original draft, Writing—review & editing. AA: Writing—original draft, Writing—review & editing. VB: Writing—original draft, Writing—review & editing. AD: Writing—original draft, Writing—review & editing. FD: Writing—original draft, Writing—review & editing. MSa: Writing—original draft, Writing—review & editing. MSe: Writing—original draft, Writing—review & editing.
